# Account suspended: Insufficient empathy

**DOI:** 10.1017/S1478951526103241

**Published:** 2026-07-17

**Authors:** Zongyu Li

**Affiliations:** East China Normal University, China

Each intake call, I used to fill the silence
with a warm, professional voice –
now I measure it in units –
fifteen-minute slots, boxes ticked
on a screen that never weeps.

They trained me to hold the trauma
of others like a newborn.
But the babies kept coming,
underfed, bruised.
My arms were audited for efficiency.

My supervisor says,
“Practice self-care, so you can care for others.”
I buy a candle, a yoga app.
The scent of lavender
mocks the mould in the client’s flat.
Self-care greases the burnout machine,
keeps it whispering.

Yesterday I looked into a woman’s eyes
and felt nothing.
No – I felt something
worse than nothing: relief.
Relief that her crisis would end on someone else’s shift.
That was the moment my empathy
declared bankruptcy.

But the system doesn’t let you go offline.
It sends automated reminders:
“Your compassion inventory is low. Refill now
with this webinar on resilience.”
I didn’t click.

Now I sit in the staff room,
not crying, not smiling.
I am learning to un-care
with precision.
What I mistook for numbness
is the first cold breath
of a pent-up rage.
I am no longer a sponge
soaking up the spill of a frayed system.
I am a witness,
and one day, a plaintiff.

Outside the window, a streetlight flickers,
holds, flickers again.
The staff room clock hums.
Somewhere a kettle boils, someone laughs.
The screen on my desk
still blinks with unread reminders.
I close the folder.
The silence is not empty –
it has begun to fill.

## Data Availability

The data that support the findings of this study are available upon request from the corresponding author. The data are not publicly available due to privacy or ethical restrictions.

